# Factors Associated With Foot Complications Among Individuals With Type 2 Diabetes Mellitus in Semi‐Urban Udupi District

**DOI:** 10.1002/edm2.70169

**Published:** 2026-03-31

**Authors:** G. Arun Maiya, Shubha Hebbar, Prabhath Kalkura, J. Vennila, Tina Agnes, Shetty Shrija Jaya, Manjunath Hande, Shashikiran Umakanth, K. N. Shivashankara, B. Ananthakrishna Shastri, David G. Armstrong, Ashu Rastogi, Neil Reeves, U. Anushree

**Affiliations:** ^1^ Department of Physiotherapy Manipal College of Health Professions, Manipal Academy of Higher Education Manipal India; ^2^ Lutheran Homes Group Gynde South Australia Australia; ^3^ UNICEF Hyderabad Field Office for Andhra Pradesh, Karnataka and Telangana Hyderabad Telangana India; ^4^ Faculty of statistics Manipal College of Health Professions, Manipal Academy of Higher Education Manipal India; ^5^ Department of Medicine Kasturba Medical College, Manipal Academy of Higher Education Manipal India; ^6^ Department of Medicine Dr. TMA Pai Hospital Udupi India; ^7^ Manipal Academy of Higher Education Manipal India; ^8^ Southwestern Academic Limb Salvage Alliance, Department of Surgery, Keck School of Medicine University of Southern California Los Angeles USA; ^9^ Department of Endocrinology and Metabolism PGIMER Chandigarh India; ^10^ Lancaster Medical School, Faculty of Health and Medicine Lancaster University Lancaster UK

**Keywords:** diabetic foot, India, logistic models, type 2 diabetes mellitus

## Abstract

**Objectives:**

Diabetic foot disorders continue to be among the most prevalent and overlooked complications associated with diabetes. The aim of this study was to determine the factors associated with diabetic foot complications in semi‐urban Udupi District.

**Methods:**

The study was a cross‐sectional study. 25,000 individuals living in Udupi district were screened for *diabetes mellitus*, and among them, 3844 individuals were found to have *type‐*2 *diabetes mellitus (T2DM)*. Further, detailed anthropometry and foot assessments were performed for these individuals.

**Results:**

In this study, a total of 3844 participants aged between 40 and 75 years with T2DM were screened to determine the prevalence of foot complications. The mean age of the study participants was 59.2 years (±11.7). Of the participants, 41.3% were male and 58.7% were female. Neuropathy was present in 9.8% of the participants, and 5.6% of the participants had a foot ulcer. Among 3844 individuals, sensation, pedal pulse, vibration, and foot care awareness were factors associated with diabetic foot complications. The Bayesian Neural Network (BNN) model was also developed, and showed good predictive performance, with an AUC of 0.901 for the right foot and 0.922 for the left foot. The BNN results also show strong predictive performance. Both models predicted diabetic foot complications.

**Conclusion:**

Prevalence of foot complications is high in the Udupi district, and the presence of risk factors puts the individual at risk for serious complications of T2DM.

## Graphical abstract

A cross‐sectional investigation conducted in semi‐urban Udupi involving 25,000 participants revealed a T2DM prevalence of 3844 individuals. The study noted a high incidence of neuropathy and ulcers, with identified risk factors including diminished sensation, absent pedal pulse, vibration impairment, and inadequate foot‐care knowledge. A Bayesian Neural Network demonstrated efficacy in predicting complications, thereby indicating potential for early identification and intervention strategies.

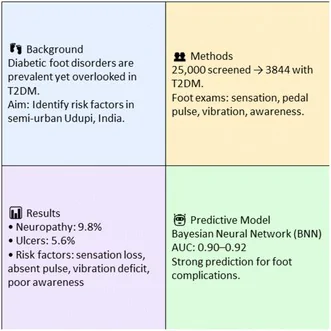

## 1 Introduction

*Diabetes Mellitus* (DM) is a highly prevalent non‐communicable disease with an estimated global prevalence of 537 million in 2021. It is projected that this figure will rise to 783 million by the end of 2045 [1]. In 2019, India had 77 million individuals with Type 2 Diabetes Mellitus (T2DM), which is expected to rise by 134 million by 2045 [2]. The alarming aspect of T2DM in India is the extensive complications that impose a substantial healthcare burden on families and individuals. T2DM is associated with several microvascular and macrovascular complications [3]. Among them, the most common complications seen in the Indian population are diabetic neuropathy (24.6%), cardiovascular complications (23.6%), retinopathy (16.6%), renal issues (21.1%), and foot ulcer (5.5%). The severity of these complications correlates with the duration of the disease. Poor glycemic management among the Indian diabetic population leads to these microvascular and macrovascular changes [4, 5].

Consequently, due to these complications, there is an increase in health care expenditures associated with the treatment of complications arising from Diabetes. Among all the complications, a minimum of 15% of the total diabetic population will experience lower limb complications over the course of their lifetime [6]. The World Health Organization categorises all foot‐related complications experienced by individuals with diabetes, such as diabetic foot syndrome. This syndrome is characterised by foot ulceration accompanied by neuropathy and varying levels of reduced blood flow and infection [7, 8]. Foot ulceration is widely recognised as a dangerous complication of diabetes, as it contributes to at least 80% of the lower‐limb amputations [6]. India faces significant public health challenges stemming from the rising prevalence of diabetes and its associated complications. With the high prevalence of diabetic foot complications and associated mortality rates across India, there is a significant need for localised research and intervention to potentially mitigate the projected risk of such complications in the coming years. The burden of diabetic foot issues is particularly acute among the rural and semi‐urban populations of the country, accounting for over 51.8% of cases [9].

The factors or predictors of foot complications in people living with T2DM include long term diabetes, poor glycemic control, male sex, older adults, peripheral neuropathy and arterial disease, hypertension, high blood glucose levels, altered plantar pressure distribution, and foot care practices [10, 11, 12, 13]. These identified factors can vary across different populations, ethnic groups and settings. Additional studies are required to identify various modifiable risk factors for predicting and preventing diabetic foot complications [14, 15]. There is also a dearth of literature on the factors associated with diabetic foot complications among rural and semi‐urban communities. Therefore, this study aims to determine the factors associated with diabetic foot complications in a rural and semi‐urban Udupi community. The findings of this study will strengthen the existing literature on community‐based studies.

## 2 Methods

### 2.1 Ethical Considerations

The study was conducted after the approval by The Kasturba Medical College and Kasturba Hospital Institutional Ethics Committee (IEC 700/2015). The study was funded by the “Diabetic Foot Care‐Stepping Ahead” (WDF 15‐941) sponsored by the World Diabetes Foundation (WDF). To cover the larger sample size, Accredited Social Health Activists (ASHA) were trained by the World Diabetes Foundation team. ASHAs are community health workers that were established as part of the National Rural Health Mission (NRHM) by the Ministry of Health and Family Welfare (MoHFW) of the Indian government. The details of the training have been mentioned in another study [10]. Written informed consent was obtained from all participants prior to their inclusion in the study. All participants identified with foot complications were provided with diabetic foot care education and, depending on the severity and condition, were referred to the outpatient departments of Kasturba hospital.

### 2.2 Study Design and Samples

This was a cross‐sectional study, and the samples were recruited from the rural and semi‐urban parts of Udupi district of Karnataka. Udupi District is located in the southern coastal region of India towards the west coast of Karnataka and has seven taluks. As per the last census, which was carried out in 2011, the total population of Udupi district was 11,77,361, out of which 28.37% lived in urban regions, and 71.63% lived in rural regions. Therefore, the focus for the present study was on rural and semiurban areas. Out of 25,000 total screenings (individuals aged 40–75 years), 3844 with diabetes were assessed for the prevalence of foot complications and associated risk factors. To ensure fair selection when multiple eligible individuals were present at community diabetic foot screening events, the Kish Grid method was utilised. This method, typically used in household surveys, can be adapted for community settings by considering groups of eligible participants at a camp or cluster as the unit for selection. Each adult with diabetes attending a camp was assigned a sequential number. A random selection was then made from the Kish Grid database using the camp identification number to determine who would undergo a foot examination. This method ensured every eligible candidate had an equal opportunity for selection, thereby minimising interviewer bias, preventing the over‐selection of readily available groups, and enhancing the representativeness of the community sample. By consistently applying the Kish Grid across all sites, the study's risk factor profile and prevalence estimates for diabetic foot issues within the target population became more accurate. Further, the following physical parameters were assessed in the diabetic patients:

### 2.3 Anthropometric Measurements and Demographic Characteristics of Participants

The study participants underwent a series of physical assessments, including anthropometric measurements of their stature, body mass, and waist circumference. Furthermore, their blood pressure was evaluated while the participants were seated, utilising a digital sphygmomanometer device on their right upper limb (Omron HEM‐7121).

### 2.4 Random Blood Sugar (RBS)

The random blood glucose levels were measured using Accu‐check Performa or Accu‐check Performa‐Nano glucometers. The next day, ASHA personnel performed fasting blood glucose testing on individuals who had a random blood glucose level of more than 140 mg/dL but no history of T2DM. For a thorough foot examination and additional study, those with fasting blood glucose levels of 126 mg/dL or higher were included. Additionally, the presence of foot problems was assessed in patients with pre‐existing T2DM who were on medication or insulin therapy.

### 2.5 Diabetic Foot Assessment

The physical exam of the foot documented musculoskeletal issues like dry skin, calluses, bunions, toe deformities, fissures, ulcers, and infections. The vascular assessment focused on palpating the pedal pulse in the dorsalis pedis or posterior tibial arteries, reporting it as present or absent. Neuropathy was evaluated through sensory tests, assessing light touch with cotton, protective sensation with a 10‐g Semmes Weinstein monofilament, and vibratory sensation with a 128‐tuning fork.

The study comprehensively evaluated participants, collecting detailed medical histoy, data on physical activity levels, information on comorbidities and risk factors, details on the type of footwear used, assessment of foot care knowledge which is defined as a broad range of therapeutic, educational, and preventive measures used to safeguard the foot and ankle from harm, encourage self‐care behaviours, and control risk factors to avoid ulceration, infection, and amputation [16].

### 2.6 Statistical Analysis

Statistical analyses were performed using R and Jamovi 2.5.3. Kolmogorov–Smirnov test was used to check the normality. The continuous data were presented as means ± standard deviation since it followed the normality assumption, and categorical variables were presented as percentages and frequencies. Binomial Logistic Regression was performed to evaluate predictive performance and assess its suitability for capturing the complexities of the dataset. It provides predictions as an interpretable baseline, with performance compared using metrics based on accuracy, AUC, and other metrics. Statistical significance was considered at *p* < 0.05.

## 3 Results

### 3.1 Demographic Characteristics of Participants

In this study, a total of 3844 participants aged between 40 and 75 years with T2DM were screened to determine the prevalence of foot complications. The mean age of the study participants was 59.2 years (±11.7) with 1588 (41.3%) of male and 2256 (58.7%) of female, an average height of 155 cms (±10.1), and weight of 60.4 kgs (±60.4). The mean RBS level was 211 mg/dL (±87.8) (Table 1). The above information is highlighted in the baseline information of T2DM in the Udupi district.

**TABLE 1:** Demographic details of T2DM of Udupi district.

Baseline characteristics	Mean ± SD / %
Age (years)	59.2 ± 11.7
Gender (*n*/%)	Males = 1588 (41.3%) Females = 2256 (58.7%)
Height (cm)	155 ± 10.1
Weight (kg)	60.4 ± 17.7
RBS (mg/dL)	211 ± 87.8

### 3.2 Self‐Reported Co‐Morbidities Among People Living With T2DM

From the Table 2, it is observed that 81.1% had no co‐morbidities, while the most common co‐morbidities included hypertension (9.5%) and eye problems (5.7%).

**TABLE 2:** Co‐morbidities with T2DM.

Co‐morbidities	Frequency (%)
Eye problems	220 (5.7%)
Heart problems	66 (1.7%)
HTN	365 (9.5%)
Kidney problems	10 (0.2%)
Brain	3 (0.1%)
Obesity	64 (1.7%)
No co‐morbidities	3116 (81.1%)

### 3.3 Prevalence of Various Risk Factors Among People With T2DM

Table 3 presents the distribution of key risk factors among the study participants. Exercise adherence defined as the degree to which an individual consistently participates in a prescribed exercise program in terms of frequency, intensity, duration, and type, as recommended by healthcare or exercise professionals [17] was reported in 21.2% (*n* = 814) of individuals, while 78.8% (*n* = 3030) did not adhere to regular exercise. *F*oot care knowledge was observed in 47.4% (*n* = 1822) of participants, whereas 52.6% (*n* = 2022) lacked proper knowledge of foot care practices. Smoking was found to be relatively rare in the study population, with only 0.5% (*n* = 18) of individuals reporting smoking habits, while the vast majority, 99.5% (*n* = 3826), were non‐smokers.

**TABLE 3:** The prevalence of various risk factors among patients with T2DM.

Risk factors	Present (*n*{%})	Absent
Exercise adherence	814 (21.2%)	3030 (78.8%)
Foot care knowledge	1822 (47.4%)	2022 (52.6%)
Smoking	18 (0.5%)	3826 (99.5%)

### 3.4 Prevalence of Foot Complications Among People Living With T2DM

Table 4 presents the distribution of foot complications observed among the study participants, categorised into musculoskeletal, vascular, and neuropathic complications. Among musculoskeletal complications, the most frequently reported condition was fissures (20.3%, *n* = 782), followed by complications of ingrown nails (17.4%, *n* = 668), callus formation (14.2%, *n* = 546), and toe deformities (13.6%, *n* = 521). Bunions were observed in 7.3% (*n* = 281) of participants. In terms of vascular complications, 5.9% (*n* = 227) of individuals exhibited absent pedal pulses, indicating possible circulatory impairment. Among neuropathic complications, 9.8% (*n* = 378) of participants were diagnosed with neuropathy, while 7% (*n* = 271) had bacterial or fungal infections. The presence of ulcers was noted in 5.6% (*n* = 216) of the population, highlighting the severity of foot complications in individuals with T2DM.

**TABLE 4:** Prevalence of foot complications among people living with T2D.

Parameter	Foot complications	Present (%)
Musculoskeletal complications	Fissures	782 (20.3%)
	Bunion	281 (7.3%)
	Callus	546 (14.2%)
	Toe deformity	521 (13.6%)
	Ingrown nails	668 (17.4%)
Vascular complications	Absent pedal pulse	227 (5.9%)
Neuropathic complications	Neuropathy	378 (9.8%)
	Infection (bacterial, fungal)	271 (7%)
	Ulcer	216 (5.6%)

### 3.5 Key Predictor Factors for Loss of Protective Sensation

Table 5 presents the results of binomial logistic regression along with their estimates, odds ratios, *p*‐values, and 95% confidence intervals for key predictor factors associated with monofilament right foot and left foot outcomes. This analysis showed that many factors were significantly associated with loss of protective sensation. For the right foot, absence of pedal pulse (OR = 4.89, *p* < 0.001), abnormal right tuning fork test (OR = 16.75, *p* < 0.001), and absent sensation (OR  = 2.63, *p* = 0.037) were significantly associated with increased odds of loss of protective sensation with respect to normal. The substantial predictors for prediction of left foot ulcers were abnormal tuning fork result (OR = 12.91, *p* < 0.001) and absent sensation (OR = 5.32, *p* < 0.001) and abnormal pedal pulse (OR = 2.46, *p* = 0.019). Loss of protective sensation can be predicted by vascular and sensory examinations. Participants who reported engaging in exercise had significantly lower odds of loss of protective sensation on the left foot (OR = 0.53, *p* = 0.001) and physical activity was associated with increased odds of a normal test result on the left foot (OR = 1.40, *p* = 0.046), signifying a potential benefit, though the clinical relevance of this association may be limited due to the small effect size. The presence of a bunion on the left foot was significantly associated with increased odds of loss of protective sensation (OR = 4.01, *p* = 0.042). Other deformities like toe deformities and callus showed elevated odds ratios but were not statistically significant. Importantly, some variables such as infection of the left foot (OR = 4.46, *p* = 0.173) and ulcer (OR = 3.09, *p* = 0.255) showed large odds ratios but did not reach statistical significance, likely due to wide confidence intervals and small subgroup sizes. The presence of a bunion on the left foot was significantly associated with higher odds of loss of protective sensation (OR = 4.01, *p* = 0.042). Other deformities such as toe deformities or callus had higher odds ratios but were not statistically significant. From the above results, it is observed that the McFadden *R* ^2^ values of left and right were 0.509 and 0.494, respectively; it is indicating a substantial proportion of variance explained by the model. The above findings emphasise the relevance of peripheral vascular status, sensation, and structured foot assessments in identifying individuals at risk for neuropathy. So, the model demonstrated good predictive performance, with an AUC of 0.920 for the right foot and 0.909 for the left foot.

**TABLE 5:** Association between key predictor factors on Monofilament testing.

Variables	Reference	Predictor	Monofilament right feet					Monofilament left feet				
			Estimate	Odds ratio	*p*	95% Confidence interval		Estimate	Odds ratio	*p*	95% Confidence interval	
						Lower	Upper				Lower	Upper
Age			0.0002	1.0000	0.9830	0.9854	1.0200	0.0012	1.0010	0.8760	0.9869	1.0160
Gender	Male	Female	0.0136	1.0140	0.9420	0.6999	1.4700	0.0183	1.0180	0.9210	0.7106	1.4600
Diabetes mellitus type	1	2	0.2695	1.3090	0.4470	0.6533	2.6200	−0.0457	0.9550	0.9000	0.4702	1.9410
Height			−0.0087	0.9910	0.3680	0.9726	1.0100	−0.0007	0.9990	0.9340	0.9807	1.0180
Weight			0.0011	1.0010	0.8720	0.9877	1.0100	0.0022	1.0020	0.7610	0.9879	1.0170
Waist circumference	0	10	−0.1108	0.8950	0.6180	0.5791	1.3800	−0.2107	0.8100	0.3320	0.5294	1.2390
	0	20	−0.1034	0.9020	0.6350	0.5882	1.3800	−0.1496	0.8610	0.4860	0.5651	1.3120
	0	30	−0.9544	0.3850	0.1950	0.0909	1.6300	−0.9276	0.3950	0.1840	0.1008	1.5520
Physical activity	0	10	−0.9817	0.3750	0.1860	0.0875	1.6000	−1.1331	0.3220	0.1050	0.0818	1.2670
	0	20	−0.0984	0.9060	0.7210	0.5284	1.5500	−0.4213	0.6560	0.1160	0.3883	1.1090
	0	30	−0.2787	0.7570	0.1920	0.4979	1.1500	−0.4350	0.6470	0.0420	0.4253	0.9850
Duration of diabetes	0	1	−0.2899	0.7480	0.1500	0.5045	1.1100	−0.3140	0.7310	0.1110	0.4968	1.0740
	0	2	−0.2203	0.8020	0.4210	0.4693	1.3700	−0.4200	0.6570	0.1040	0.3961	1.0900
	0	3	−0.0315	0.9690	0.9220	0.5160	1.8200	−0.2053	0.8140	0.5040	0.4458	1.4880
Management	0	1	−0.5504	0.5770	0.2850	0.2105	1.5800	−0.6026	0.5470	0.2390	0.2007	1.4930
	0	2	1.4367	4.2070	0.2880	0.2978	59.4400	0.0958	1.1000	0.9340	0.1144	10.5860
Exercise	No	Yes	−0.3188	0.7270	0.1260	0.4832	1.0900	−0.6322	0.5310	0.0010	0.3611	0.7820
Physical activity	No	Yes	0.2003	1.2220	0.2510	0.8679	1.7200	0.3369	1.4010	0.0460	1.0058	1.9510
History of foot ulcer	No	Yes	0.3192	1.3760	0.5980	0.4205	4.5000	−0.2758	0.7590	0.6120	0.2618	2.2000
Dry skin colour changes right	No	Yes	−0.9890	0.3720	0.1670	0.0915	1.5100	−0.4751	0.6220	0.4460	0.1833	2.1090
Dry skin colour changes left	No	Yes	0.6563	1.9280	0.3630	0.4689	7.9300	0.2336	1.2630	0.7100	0.3679	4.3370
Toe deformities right	No	Yes	−0.7179	0.4880	0.2550	0.1418	1.6800	−0.3152	0.7300	0.6160	0.2127	2.5030
Toe deformities left	No	Yes	0.3920	1.4800	0.5430	0.4186	5.2300	0.0157	1.0160	0.9800	0.2916	3.5390
Ingrown nails right	No	Yes	2.6744	14.5030	0.2760	0.1178	1785.1200	0.8608	2.3650	0.6070	0.0888	63.0040
Ingrown nails left	No	Yes	−2.0871	0.1240	0.3940	0.0010	15.1300	−0.2398	0.7870	0.8830	0.0321	19.3150
Bunions right	No	Yes	−0.4079	0.6650	0.4210	0.2461	1.8000	0.3283	1.3890	0.5340	0.4937	3.9060
Bunions left	No	Yes	1.3891	4.0110	0.0420	1.0513	15.3000	0.6695	1.9530	0.3140	0.5306	7.1910
Infection right	No	Yes	−1.1484	0.3170	0.4180	0.0197	5.1100	−2.2617	0.1040	0.0360	0.0126	0.8640
Infection left	No	Yes	0.1527	1.1650	0.9150	0.0710	19.1000	1.4945	4.4570	0.1730	0.5184	38.3220
Callus right	No	Yes	2.3213	10.1890	0.0530	0.9728	106.7200	1.6103	5.0040	0.1620	0.5230	47.8800
Callus left	No	Yes	−2.2606	0.1040	0.0590	0.0100	1.0900	−1.7470	0.1740	0.1280	0.0183	1.6570
Fissure right	No	Yes	−0.4161	0.6600	0.7630	0.0442	9.8600	−0.1364	0.8730	0.9010	0.1022	7.4500
Fissure left	No	Yes	−0.2792	0.7560	0.8400	0.0503	11.3800	−0.3335	0.7160	0.7620	0.0831	6.1760
Ulcer right	No	Yes	−1.5162	0.2200	0.2430	0.0172	2.8000	−1.6592	0.1900	0.1030	0.0259	1.3970
Ulcer left	No	Yes	1.4189	4.1320	0.2710	0.3300	51.7500	1.1295	3.0940	0.2550	0.4422	21.6530
Sensation right	Absent	Normal	0.9677	2.6320	0.0370	1.0588	6.5400	0.0614	1.0630	0.8910	0.4415	2.5610
Sensation left	Absent	Normal	0.7980	2.2210	0.0970	0.8650	5.7000	1.6720	5.3230	< 0.001	2.2796	12.4280
Right pedal pulse	Absent	Normal	1.5879	4.8930	< 0.001	2.2031	10.8700	0.6125	1.8450	0.1090	0.8728	3.9000
Left pedal pulse	Absent	Normal	−0.2885	0.7490	0.5020	0.3226	1.7400	0.8998	2.4590	0.0190	1.1629	5.2000
Left tuning fork	Absent	Normal	0.8815	2.4150	0.0080	1.2637	4.6100	2.5581	12.9120	< 0.001	7.0067	23.7940
Foot care knowledge	Absent	Normal	0.2923	1.3400	0.1040	0.9421	1.9000	0.0696	1.0720	0.6890	0.7627	1.5070
Right tuning fork	Absent	Normal	2.8185	16.7520	< 0.001	8.8828	31.5900	1.0214	2.7770	0.0010	1.4794	5.2130

### 3.6 Bayesian Neutral Network

**FIGURE 1:**
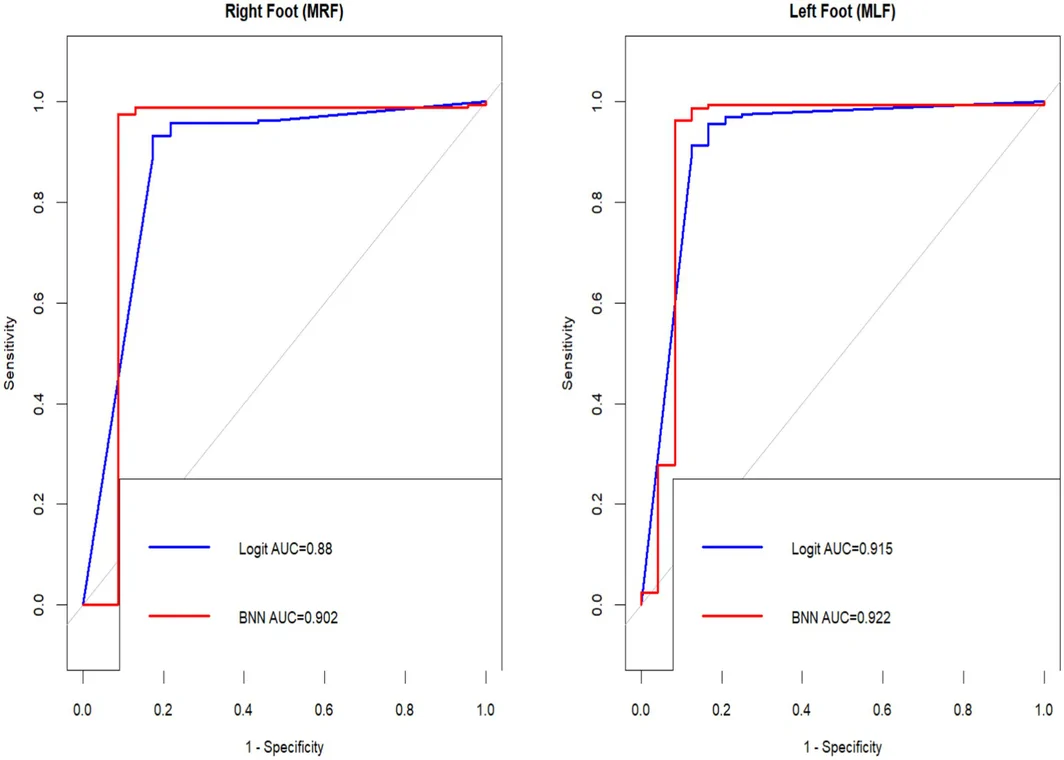
ROC curves of logistic regression and Bayesian Neural Network models for loss of protective sensation in the right and left foot with corresponding AUC values.

We constructed a Bayesian Neural Network (BNN) method for predicting diabetic foot complications that was developed using a training and testing methodology. We separated our dataset into a training set (70%) to develop the model, and a hold‐out test set (30%) for independent assessment. We performed 10‐fold cross‐validation in our training set for parameter tuning and overfitting check. The model was trained on nine folds and validated on the last fold. Cases with missing values for those model variables deemed critical to model performance were excluded. Logistic regression models were also developed on the same training set, adjusting for the other predicted variables (e.g., age, duration of diabetes etc.) as possible confounding variables. Model performance was evaluated on the test set using areas under the receiver operator curve (AUC), sensitivity and specificity. The BNN achieved AUC values of 0.902 for the right foot, 0.922 for the left foot (Table 6). Overall discrimination was slightly worse than that seen for the logistic regression, but good specificity can be maintained, which illustrates good predictive ability and potential clinical utility for early intervention. The ROC analysis demonstrated strong discriminative ability of both models in identifying loss of protective sensation. As shown in Figure 1, the Bayesian Neural Network achieved higher AUC values compared to logistic regression for both the right foot (0.902 vs. 0.88) and the left foot (0.922 vs. 0.915).

**TABLE 6:** Predictive performance of logistic regression vs. BNN (AUC, sensitivity, specificity).

Model	Foot	AUC	Sensitivity	Specificity
LR	RF	0.928	0.857	0.826
LR	LF	0.911	0.872	0.834
BNN	RF	0.901	0.988	0.871
BNN	LF	0.922	0.988	0.875

The Bayesian Neural Network (BNN) model is a relatively simple and compact design, comprising 36 nodes and 75 arcs. The model demonstrates an average Markov blanket size of 5.56 and an average neighbourhood size of 4.17, suggesting that each node is connected to only a few other nodes. With an average branching factor of 2.08, it indicates that each node has only a limited number of children. The model is optimised and achieves a favourable Bayesian Information Criterion (BIC), indicating a strong fit with the data and effective predictive performance. The penalization coefficient of 4.119929 suggests moderate regularisation, which enables the model to fit the training data well while preventing overfitting. Overall, this BNN model is well‐designed and trained, making it suitable for accurate predictions.

By using both methodologies, the study benefits from the strengths of each approach. Logistic regression offers simplicity, interpretability, and strong performance with linear data relationships, while the BNN adds the ability to model more complex patterns and account for uncertainty in predictions. This combined approach ensures a robust and comprehensive analysis, leveraging the strengths of both methods to achieve high performance and reliability in predictions.

## 4 Discussion

To the best of our knowledge, this is the first large study conducted at the level of the community to determine the prevalence of foot complications in T2DM patients. The neurological, musculoskeletal, and vascular components of foot complications were considered equally. In our study, we have found that the crude prevalence of musculoskeletal foot complications was 72.8%, vascular complications was 18.5%, and neuropathy was 9.8%. Ulcers and infections are considered as secondary complications of neuroischemic changes. In our study, the prevalence of infection was 7% and that of ulcers was 5.6% in the rural and semiurban Udupi community.

The recommended daily step count for a typical adult is approximately 7500–10,000 steps. This target may be higher in both urban and rural areas of India. Maintaining strong, healthy feet is crucial to achieve this daily goal. In individuals with diabetic foot, neuropathic joint degeneration can impact the weight‐bearing joints. Repetitive stress may lead to ligament laxity and bone fragmentation. This study examined various foot conditions, including fissures, bunions, calluses, toe deformities, and ingrown nails. Fissures were the most prevalent, while bunions were the least common. A hospital‐based study in rural northern India documented a 10.4% prevalence of diabetic foot, with 54.6% experiencing callosities and 28% exhibiting deformities among individuals with type 2 diabetes. A multi‐centric study on the urban Indian population revealed a prevalence range of 0.5%–7.5% for these conditions [18].

Alterations in pedal pulse provide a limited understanding of future vascular complications. Our study found that 18.5% of participants experienced vascular complications. Previous research has demonstrated that the incidence of peripheral vascular diseases and neuropathy is lower among Southeast Asians compared to Europeans, which could be attributed to the lower smoking prevalence [19]. The prevalence of smoking in our study was 0.5%. A hospital‐based study in southern Indian urban populations reported a 3.6% prevalence of peripheral vascular disease in individuals with T2DM [20]. Another study from an urban Indian population documented a 5% prevalence of PVD. These findings do not align with our results, as we employed broad criteria to diagnose vascular complications.

A typical side effect of T2DM is neuropathy, which can impair functioning. A common manifestation of this illness is gradual loss of sensation. Foot numbness and burning are among the symptoms. Neuropathy that develops gradually could go years without being noticed [21]. We used intact tactile, protection, and vibratory sensations to screen for neuropathy in this investigation. It was thought that a lack of protective feeling was indicated by insensitivity to a 10 g monofilament at any location on the foot. From our previous study, we noted that neuropathy in the rural Udupi taluk was 30.2%. According to a different study, neuropathy was present in 26.1% of South Indians living in cities [22, 23].

Similarly, diabetic foot infection is also a prevalent condition among individuals with T2DM, often leading to hospital admissions. A comparative analysis of rural and urban populations revealed a higher prevalence of foot complications in rural areas, reaching up to 34%. A study in the rural Udupi community found that 26.7% of individuals with T2DM had foot infections. In contrast, the prevalence of foot infections in urban Indian populations was reported to range from 6% to 11% [13, 20]. Structural abnormalities of the foot, including flatfoot, hallux valgus, claw toes, Charcot neuroarthropathy, and hammer foot, are significant contributors to the development of diabetic foot ulcers. These deformities lead to abnormal pressure on the soles of the feet and increase the risk of ulcer formation [24]. A thin layer of skin and subcutaneous tissue can lead to the formation of ulcers on the back or top of the toe, particularly in areas with abnormal foot pressure. This increased pressure can result in damage to local tissues, causing inflammation and ultimately leading to ulceration [25, 26]. The assessment of foot abnormalities is important for gaining insight into the onset, progression, and outlook of individuals with diabetic foot issues. Our study showed that loss of sensations and pedal pulse can also increase the individual at higher risk of foot complications in individuals with T2DM. Peripheral artery disease and loss of sensations hinder the process of wound healing in the lower extremities and are significant contributing factors to diabetic foot ulcers, often resulting in amputation [27].

Diabetic foot ulcers represent another significant complication. This study found a prevalence of ulcers at 5.6%, which aligns with the range of 5.3% to 10.5% reported in prior research [10, 22]. Similarly, a hospital‐based investigation in northern India documented a higher prevalence of 14.3% [23]. Importantly, this study also revealed that the occurrence of ulcers was more prevalent among individuals lacking knowledge of proper foot care and appropriate footwear.

Our results also indicated that among several factors such as exercise, structured foot assessments, peripheral vascular status, history of foot ulcers, skin texture, deformities, ingrown nails, bunions, infections, callus, fissure, ulcers, pedal pulse, and sensation, the peripheral vascular status, sensation, and structured foot assessments are the key predictors for identifying the risk for neuropathy among T2DM individuals.

### 4.1 The Strength of the Study

The accomplishment of a comprehensive community‐level examination is the study's main strength. In order to ascertain the prevalence of different foot problems in people with T2DM, this study was carried out on a representative population sample. Additionally, using the vast amount of data they had gathered, the researchers created logistic regression models.

### 4.2 Clinical Implications of the Study

Our findings highlight the urgent need for community‐level screening and intervention strategies for diabetic foot care. Training ASHAs in structured screening protocols and integrating foot assessments into primary health policies can improve early detection and timely referrals. Strengthening structured foot‐care interventions, including education and affordable footwear, will reduce complications and support long‐term diabetes management in rural and semi‐urban populations.

### 4.3 Limitations of the Study

We were unable to distinguish between motor and sensory neuropathy in a scientific manner because the investigation was carried out at the community level. As a result, we advise future research to use advanced and validated diabetic foot instruments for additional assessment. Therefore, utilising tools at the community level, this study might be carried out in a more scientific way in the future.

## 5 Conclusion

We conclude that the prevalence of foot complications is high in Udupi district, and the presence of risk factors puts the individual at risk for serious complications of T2DM. So, there is a strong need to increase awareness about foot care in the rural community. The early screening and management could possibly prevent serious complications.

## Author Contributions

Dr. G. Arun Maiya contributed to conceptualization, methodology, supervision, project administration, formal analysis, and writing (original draft and review and editing). Dr. Shubha Hebbar contributed to methodology, investigation, supervision, and writing (review and editing). Dr. Prabhath Kalkura contributed to methodology, investigation, supervision, resources, and writing (review and editing). Dr. Vennila J contributed to formal analysis, investigation, data curation, and writing (review and editing). Tina Agnes contributed to formal analysis, investigation, data curation, and writing (review and editing). Shetty Shrija Jaya contributed to investigation, data curation, and writing (review and editing). Dr. Manjunath Hande contributed to methodology and writing (review and editing). Dr. Shashikiran Umakanth contributed to methodology and writing (review and editing). Dr. Shivashankara KN contributed to methodology and writing (review and editing). Dr. B. Ananthakrishna Shastri contributed to methodology and writing (review and editing). Dr. David G. Armstrong contributed to writing (review and editing). Dr. Ashu Rastogi contributed to writing (review and editing). Dr. Neil Reeves contributed to writing (review and editing). Dr. Anushree U contributed to data curation, formal analysis, and writing (review and editing).

## Funding

This study is funded by the grant from World Diabetes Foundation sponsored project, ‘Diabetic Foot Care: Stepping Ahead’—WDF: 15/0941 and acknowledge the support of RailTel Corporation of India Limited.

## Ethics Statement

The Kasturba Medical College and Kasturba Hospital Institutional Ethics Committee (IEC 700/2015).

## Consent

Written informed consent was obtained from all enrolled patients.

## Conflicts of Interest

The authors declare no conflicts of interest.

## Data Availability

The analysed data sets generated during the present study are available from the corresponding author on reasonable request.
